# A Novel Approach for Direction of Arrival Estimation in Co-Located MIMO Radars by Exploiting Extended Array Manifold Vectors

**DOI:** 10.3390/s23052550

**Published:** 2023-02-24

**Authors:** Sadiq Akbar, Muhammad Sohail, Fawad Zaman, Muhammad Abdul Rehman Khan, Nopdanai Ajavakom, Gridsada Phanomchoeng

**Affiliations:** 1 Department of Mechanical Engineering, Chulalongkorn University, Bangkok 10330, Thailand; 2 School of Information Science and Technology, University of Science and Technology of China (USTC), Hefei 230026, China; 3 Department of Electrical & Computer Engineering, COMSATS University Islamabad, Islamabad 44000, Pakistan; 4 Department of Avionics Engineering, Institute of Space Technology Islamabad, Islamabad 44000, Pakistan; 5 Micro/Nano Electromechanical Integrated Device Research Unit, Faculty of Engineering, Chulalongkorn University, Bangkok 10330, Thailand; 6 Applied Medical Virology Research Unit, Chulalongkorn University, Bangkok 10330, Thailand

**Keywords:** direction of arrival estimation, flower pollination algorithm, co-located radar, MIMO systems

## Abstract

Multiple-input multiple-output (MIMO) radars enable better estimation accuracy with improved resolution in contrast to traditional radar systems; thus, this field has attracted attention in recent years from researchers, funding agencies, and practitioners. The objective of this work is to estimate the direction of arrival of targets for co-located MIMO radars by proposing a novel approach called flower pollination. This approach is simple in concept, easy to implement and has the capability of solving complex optimization problems. The received data from the far field located targets are initially passed through the matched filter to enhance the signal-to-noise ratio, and then the fitness function is optimized by incorporating the concept of virtual or extended array manifold vectors of the system. The proposed approach outperforms other algorithms mentioned in the literature by utilizing statistical tools for fitness, root mean square error, cumulative distribution function, histograms, and box plots.

## 1. Introduction

5G can be defined as next-generation emerging technology that increases the system’s capacity and has high data rates with improved quality of service [1,2]. The design and development of an antenna guarantee the successful operation of any 5G device. Multiple-input multiple-output (MIMO) systems are one of the most indispensable technologies used in 5G communication systems [3,4,5]. In recent years, MIMO radar systems have been the focus of research for practitioners, researchers, and funding agencies due to their enhanced capabilities of high resolution and improved estimation accuracy, as compared to the traditional phased array radar systems [6]. The research in the field of MIMO radar systems can broadly be divided into two cases, i.e., when the antennas in transmitters and receivers are widely separated, and in the second case, when the antennas in both transmitters and receivers are placed close to each other [7]. The second case has an obvious advantage over the first one, as it receives the reflected signals in the same target aspect, thus allowing a simple target model to be adopted [8]. Based on this, if the closely spaced antennas of the transmitter and receiver are placed far away from each other, then the system is said to be a bistatic MIMO system. Conversely, the system is referred to as monostatic or collocated if the receiver and the transmitter are placed close to each other, meaning that the direction of arrival (DOA) and direction of departure (DOD) are the same [9]. Several researchers have attempted to address the issue of DOA estimation for the co-located MIMO radar systems [10,11]. A discrete time Fourier transform method is exploited in [12] to estimate the DOA by sampling the received signals in the spatial domain; however, the resolution issues of this approach are limited due to the Rayleigh criterion. To overcome this issue, subspace-based approaches called super resolution methods are proposed in [13,14,15]. These methods work on the received signals’ covariance matrices by dividing the space into two subspaces called the signal subspace and noise subspace. The main issue with these techniques is their computational complexity, as they require a large number of snapshots. To reduce their computational complexity, compressed sensing-based methods are proposed in [16,17] that require a lower number of snapshots by exploiting the target sparsity in the spatial domain. In general, the compressive sensing methods are classified into greedy and norm-based methods, such as orthogonal matching pursuits (OMP) and stagewise OMP [18]. Furthermore, sparse Bayesian learning (SBL)-based approaches are also proposed using the prior assumption of sparse signals and can achieve excellent results at a relatively high computational cost [19]. In [20], another super resolution method is proposed for the DOA estimation of fast-moving targets that only uses a single snapshot. This method utilized the concept of virtual array geometries and achieved better results as compared to the ones discussed in [18,19,21]. However, it needs further improvement in estimation accuracy and computational complexity. 

In this paper, DOA estimation is carried out by proposing a novel approach called the flower pollination algorithm (FPA). This approach is simple in concept, easy to implement, and has the capability of solving complex optimization problems [22,23]. The received data from the far field located targets are initially passed through the matched filter to enhance the signal-to-noise ratio (SNR), and then the fitness function is optimized by incorporating the concept of virtual or extended array manifold vectors of the system. The proposed approach outperforms other algorithms mentioned in the literature [18,19,20,21], by utilizing statistical tools for fitness, root mean square error, cumulative distribution function, histograms, and box plots. All the simulations are carried out in MATLAB.

The following list details the salient features of this work:

- FPA is designed and implemented for the first time for DOA estimation with a monostatic/co-located MIMO radar system.
- A novel fitness function based on extended array manifold vectors is developed for the optimization of FPA.
- For different scenarios, the design scheme is validated.
- The scheme’s correctness is observed for very small deviations from the reference values.
- Different statistical performances, such as RMSE, box plots, CDF and histograms, are used to confirm the reliability, consistency and robustness of the proposed approach.

The overall presentation of the remaining work is managed as follows: the first part is dedicated to the introduction, which is followed by the system model in Section 2. The proposed methodology is discussed in Section 3, while the results and discussion are presented in Section 4. Section 5 summarizes the work, along with some future directions in the research area of DOA estimation.

## 2. System Model

**Figure 1:**
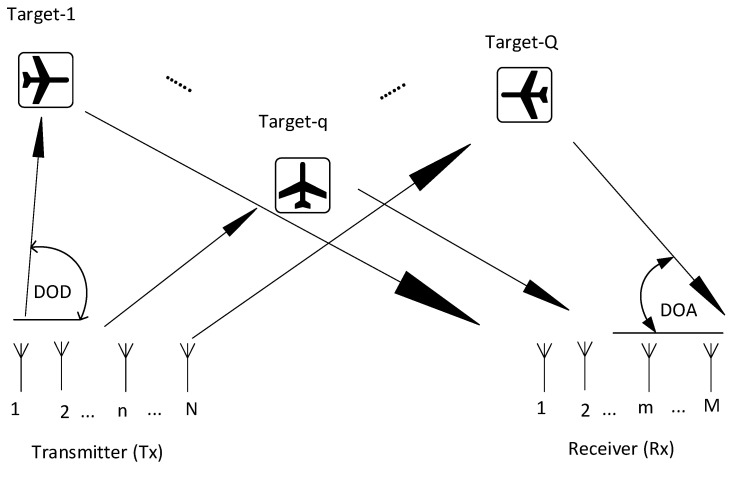
Co-Located Radar System.

In this section, a system model is developed for DOA estimation with a co-located or monostatic MIMO radar system. The transmitter (T_x_) and the receiver (R_x_) have N and M antenna sensors, respectively. The T_x_ and R_x_ are placed close enough to ensure that the DOD and DOA of the system with respect to the targets are the same, as shown in Figure 1. 

The inter-element spacings in T_x_ and R_x_ antennas are kept as ʎ/2. The T_x_ of the collocated MIMO radar transmits orthogonal signals that can be given for one pulse duration T [20] as

(1) $$\int_{0}^{T}S_{n_{1}}(t)S_{n_{2}}^{H}(t)dt=\delta (n_{1}-n_{2})$$

where $\delta (n_{1}-n_{2})=1\;for\;n_{1}=n_{2}$ and zero otherwise. It is supposed that the transmitted signals are reflected back from Q sources in space with different DOAs and are received by the R_x_ of the same collocated MIMO radar. Hence, the signal received by the m-th antenna in the R_x_ is represented as

(2) $$y_{m}(t)=\sum_{q=1}^{Q}\sum_{n=1}^{N}S_{n}(t)e^{j(Kd(n-1)\sin\psi_{q})}\;\alpha_{q}e^{j(Kd(m-1)\sin\theta_{q}}+\xi_{m}(t),\;where\;m=1,2,\ldots ,M.$$

where $\psi_{q}$ and $\theta_{q}$ represent the DOD and DOA, respectively, which are equal for the collocated MIMO radar. Similarly, K=2π/λ is the propagation constant and $\alpha_{q}$ is the reflection coefficient, while $\xi_{m}$ is the AWGN added at the m-th R_x_ antenna that has unit variance and a zero mean. Once the signal is received, it is further fed to the matched filter to improve the SNR, whose impulse response for one pulse T is $h_{n}(t)=S_{n}^{*}(T-t)$. The response of the m-th and n-th antenna in the R_x_ and T_x,_ respectively, for Q targets in space can be given as

(3) $$y_{m,n}(t)=\sum_{q=1}^{Q}\int_{0}^{T}S_{n}^{H}(t)S_{n}(t)e^{jK(n-1)d\sin\psi_{q}}\;\alpha_{q}e^{jK(m-1)d\sin\theta_{q}}\;dt+\int_{0}^{T}S_{n}^{H}(t)\xi_{m}(t)dt$$

When we incorporate Equation (1) into Equation (3), we can obtain the following equation:

(4) $$y_{m,n}(t)=\sum_{q=1}^{Q}e^{jK(n-1)d\sin\psi_{q}}\;\alpha_{q}e^{jK(m-1)d\sin\theta_{q}}+\xi_{m,n}$$

$where\;\;\xi_{m,n}=\int_{0}^{T}S_{n}^{H}(t)\xi_{m}(t)dt\;$ for a single snapshot. 

In its extended form, Equation (4) can be given as follows:

(5) $$y_{m,n}=\sum_{q=1}^{Q}\alpha_{q}g_{n}(\psi_{q})h_{m}(\theta_{q})+\xi_{m,n}\;\;$$

where $g_{n}(\psi_{q})=e^{jK(n-1)\sin\psi_{q}}\;\;and\;\;h_{m}(\theta_{q})=e^{jK(m-1)\sin\theta_{q}}\;$ for n=1,2,… N and m=1,2,…, M.

The AMV g and h for the target located in the q^th^ direction with respect to T_x_ and R_x_, can be given as

(6) $$g(\psi_{q})={\left[g_{1}(\psi_{q}),\;g_{2}(\psi_{q}),\ldots ,\;g_{N}(\psi_{q})\right]}^{H}$$

and

(7) $$h(\theta_{q})={\left[h_{1}(\theta_{q}),\;h_{2}(\theta_{q}),\ldots ,\;h_{M}(\theta_{q})\right]}^{H}$$

Now, with the signal received at the output of the matched filter for the (m,n)^th^ combination of antennas, the following equation can be used:

(8) $$y_{m,n}=\sum_{q=1}^{Q}\alpha_{q}g_{n}(\psi_{q})h_{m}(\theta_{q})+\xi_{m,n}$$

The response of the m-th R_x_ antenna for the entire T_x_ array can be given as follows:

(9) $$y_{m}=\sum_{q=1}^{Q}\alpha_{q}h_{m}(\theta_{q})g(\psi_{q})+\xi_{m}$$

For the response of the entire R_x_ array, the concept of extended array manifold vectors (EAMV) is used, which increases the dimension of our observed vector, i.e.,

(10) $$y=\sum_{q=1}^{Q}\alpha_{q}h(\theta_{q})⊗g(\psi_{q})+\xi$$

where ⊗ is the Kronekar product. Equation (10) can be given in vector form as follows:

(11) y=Bβ+ξ

where $B=[h(\psi_{1})⊗g(\theta_{1}),\;h(\psi_{2})⊗g(\theta_{2}),\ldots ,\;h(\psi_{Q})⊗g(\theta_{Q})]$ and $\beta =[\alpha_{1},\alpha_{1},\ldots ,\;\alpha_{Q}]$.

For co-located MIMO radars, the DOD (ψ) and DOA (θ) are equal, so the problem at hand is to precisely and efficiently estimate the unknown $\theta_{q}$ for q=1,2,…,Q.

## 3. Proposed Methodology

Xin-She Yang, in 2012, was inspired by the pollination process in flowers and developed an efficient algorithm called the flower pollination algorithm (FPA) [24]. Due to its ease of implementation and capabilities of solving complex optimization problems, the FPA attracted scientists and engineers from different fields of science and engineering, such as control system engineering [25], wireless networks [26], power systems [27], image processing [28], clustering and classification [29], computer gaming [30] and electrical systems [31].

In this algorithm, the concept of reproduction of new flowers in flowering plants based on the pollination process is introduced, consisting of two types, namely biotic (global pollination) and abiotic (local pollination). In both types, the pollen of one flower meets pollen from another flower that may belong to the same plant or another plant that belongs to the same species. As a result, fruitful fertilization can take place in this algorithm. The pollinators or carriers of pollen are different in both processes, i.e., insects and birds take part in the biotic pollination process, while wind or simple diffusion acts as pollinators in the abiotic process. In nature, the biotic process takes place more often than the other process. Flower constancy also plays a major role in the pollination process, due to the fact that pollinators restrict themselves to the plants of a certain species [32]. The global and local pollinations in this algorithm are given as

(12) $$x_{i}^{t+1}=x_{i}^{t}+L(x_{i}^{t}-g_{best})$$

and

(13) $$x_{i}^{t+1}=x_{i}^{t}+\varepsilon (x_{j}^{t}-x_{k}^{t})$$

In these equations, L denotes the Levy flights, which determines the pollination strength and is always greater than 0 and ε denotes the uniform distributions, respectively. 

**Figure 2:**
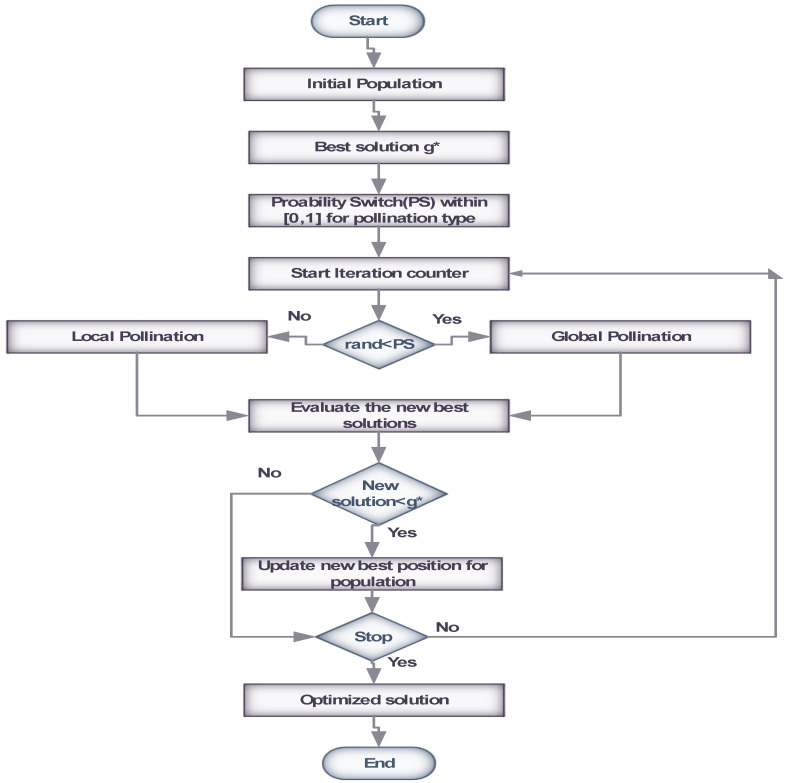
FPA Flow Chart: Where * denotes global best.

**Figure 3:**
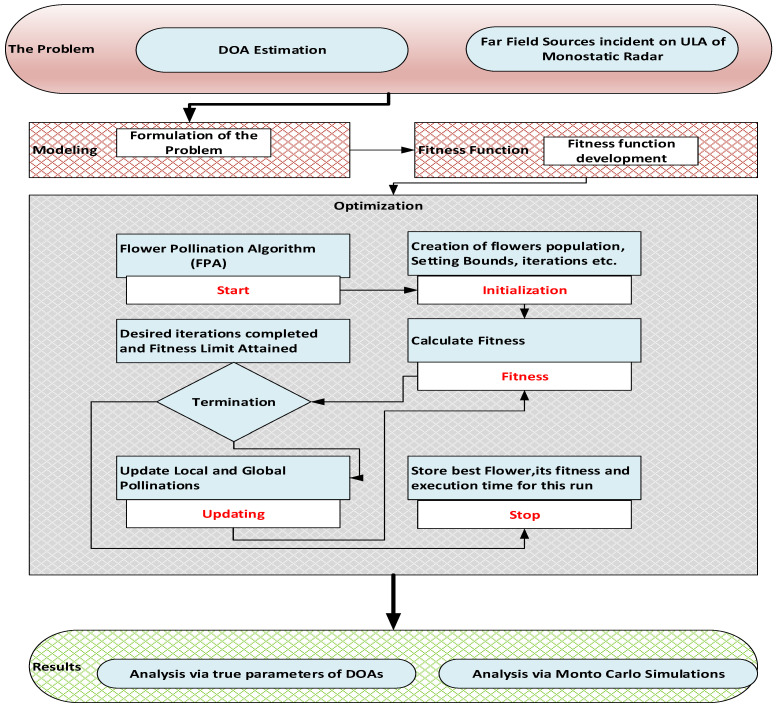
Overall Graphical Abstract.

In this work, we have designed and exploited the FPA for the estimation of the DOA in a co-located MIMO radar. In this regard, the flow diagram of FPA is shown in Figure 2, while its steps in the form of a pseudo code are given below. The overall graphical abstract of the entire work is shown in Figure 3. 

Step1: Initialization. A random population of N_p_ flowers/plants/pollens is generated. Each member of this population has as many entries as there are decision variables, i.e., the number of DOA estimations of the targets. The j-th member representation of the FPA is given as

(14) $${\hat{F}}_{j}={\left(\begin{array}{cccc} f_{1} & f_{2} & \cdots & f_{N_{p}} \end{array}\right)}^{H}={\left(\begin{array}{cccc} {\hat{\theta }}_{1} & {\hat{\theta }}_{2} & \cdots & {\hat{\theta }}_{N_{p}} \end{array}\right)}_{j}{}^{H}\;\;where\;\;\hat{\theta }=\left[{\hat{\theta }}_{1},\;{\hat{\theta }}_{2},\;\cdots ,\;{\hat{\theta }}_{Q}\right]\;$$

The constraints associated with the underlying problem are as follows: $\theta \;\in \;R\;:\;-90^{\circ }\;\;\leq \;\;\theta_{q}\;\;\leq \;\;90^{\circ }\;\;where\;q=1,\;2,\;3,\;\ldots ,\;Q$. Further settings of the FPA parameters, such as the population size, the number of iterations and the limit for probability switching, are set.

Step2: Fitness Evaluation. The flower members of the population ${\hat{F}}_{j}$ are evaluated one by one by the fitness function using the mean square error (MSE) concept, in which both the responses (desired and estimated) are based on EAM vectors and are ranked accordingly, i.e.,

(15) $$f\varepsilon =E\left({\left|B\beta -\hat{B}\beta \right|}^{2}\right)$$

In Equation (15), the array manifold matrix **B** and **β** are defined in Equation (11), where

(16) $$\hat{B}=[h({\hat{\psi }}_{1})⊗g({\hat{\theta }}_{1}),\;h({\hat{\psi }}_{2})⊗g({\hat{\theta }}_{2}),\ldots ,\;h({\hat{\psi }}_{Q})⊗g({\hat{\theta }}_{Q})]$$

Step3: The initial best member (solution) g* is obtained.

Step4: The limit for the probability switch (PSW) is defined as (0,1).

Step5: Fitness values of all N_p_ members/flowers/solutions are computed.

Step6: If rand is less than PSW, then

Step7: A step vector L of dimension d (while obeying the Levy distribution) is drawn.

Step8: Global pollination is carried out using Equation (12).

Step9: A uniform distribution ε ∈ [0,1] is drawn.

Step10: Among all the solutions, j and k are chosen randomly.

Step11: Local pollination is carried out using Equation (13).

Step12: A new best solution is evaluated.

Step13: If the evaluated new solution is less than g*,

Step14: $x^{t}=x^{t+1}$

Step15: The current best solution g* is found within all $x_{i}^{t}$.

Step16: The best solution and its fitness are stored as the global best solution for each run.

Step17: Steps 1–16 are performed multiple times to obtain a large data set for reliability purposes.

Step18: The fitness function given in Equation (15) and the RMSE given in Equation (17) are used as performance metrics for the proposed scheme.

(17) $$RMSE=\sqrt{\frac{\sum_{s=1}^{Runs}{\left\|\left(\theta_{s}-{\hat{\theta }}_{s}\right)\right\|}^{2}}{NE}},\;\;NE\;being\;number\;of\;elements\;in\;\theta$$

## 4. Results and Discussion

In this section, several simulations are carried out to validate the performance and reliability of the proposed flower pollination algorithm for the DOA estimation of targets located in the Fraunhoper zone, with respect to the R_x_ of a co-located MIMO radar system. The T_x_ and R_x_ of the co-located MIMO radar system are equipped with uniform linear arrays with N and M antennas, respectively. This section is mainly divided into two parts; in the first part, the overall performance of FPA is analyzed for different scenarios based on a different number of targets. A comparison with the state-of-the-art algorithms [18,19,20,21] is carried out in the second part. Initially, all the received signals at the R_x_ side of the monostatic MIMO radar are passed through a matched filter to improve the SNR, and then the concept of extended array manifold (EAM) vectors is used to optimize the fitness function defined in Equation (15). The inter-element distance between any two adjacent antennas is taken to be half of the wavelength on both the T_x_ and R_x_ sides. Throughout the simulations, the values of the reflection coefficients are considered as unity. All of the desired and the estimated vectors of DOAs are in degrees. The proposed scheme (FPA) requires a single snapshot to optimize the fitness function. The parameter settings of the proposed FPA are given in Table 1. All the results are taken from 100 independent trials of the proposed algorithm. The system specifications are shown in Table 2.

**Figure 4:**
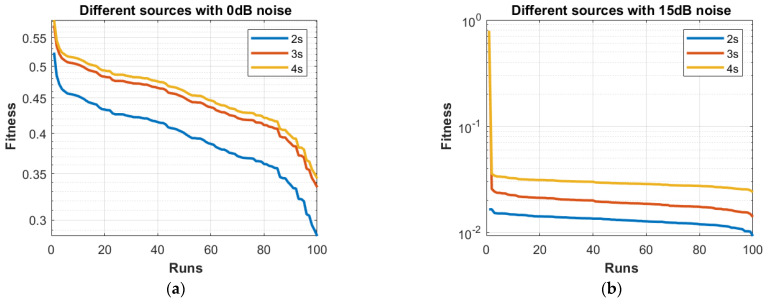
Fitness with Noises (**a**) 0 dB and (**b**)15 dB.

**Figure 5:**
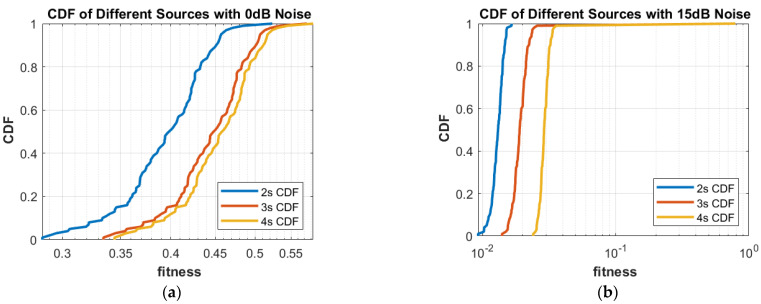
CDF with Noises (**a**) 0 dB and (**b**) 15 dB.

**Figure 6:**
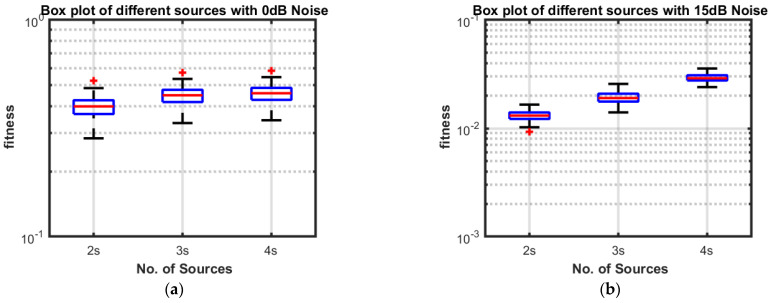
Box Plot with Noises (**a**) 0 dB and (**b**) 15 dB.

**Figure 7:**
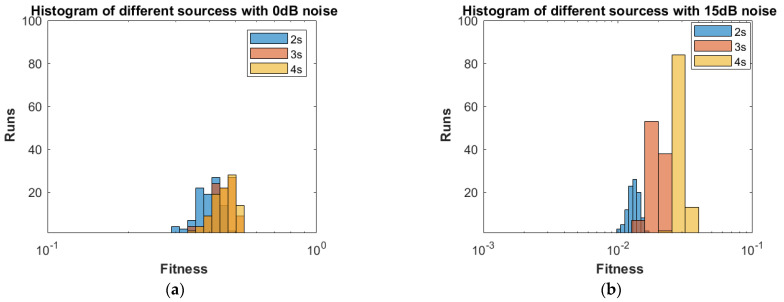
Histogram with Noises (**a**) 0 dB and (**b**) 15 dB.

**Figure 8:**
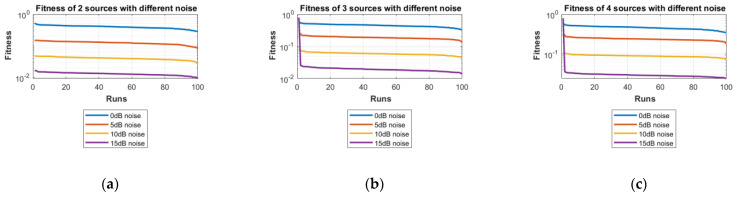
Fitness with Different Noises: (**a**) Two Sources; (**b**) Three Sources; (**c**) Four Sources.

**Figure 9:**
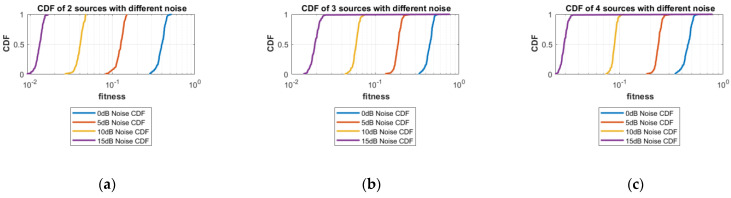
CDF with Different Noises: (**a**) Two Sources; (**b**) Three Sources; (**c**) Four Sources.

**Figure 10:**
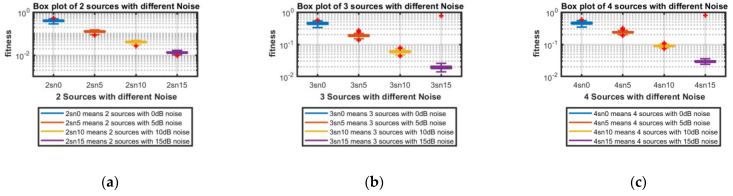
Box Plot with Different Noises: (**a**) Two Sources; (**b**) Three Sources; (**c**) Four Sources.

**Figure 11:**
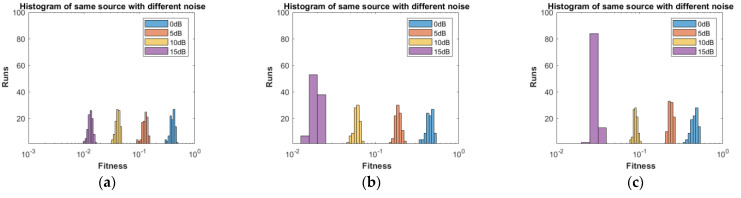
Histogram with Different Noises: (**a**) Two Sources; (**b**) Three Sources; (**c**) Four Sources.

**Part 1:** In this part, DOAs are estimated for two sources, three sources and four sources. The analysis of the data is carried out in two different scenarios, that is, the different number of targets or sources for the same level of noise, as shown in Figure 4, Figure 5, Figure 6 and Figure 7, and the same number of targets for different levels of SNR, as shown in Figure 8, Figure 9, Figure 10 and Figure 11. In the first scenario, four different cases were taken into account i.e., the 0 dB noise case, 5 dB noise case, 10 dB0 dB noise case and 15 dB noise case. The best fitness values obtained in 100 independent runs of the proposed algorithm FPA are shown with different statistical tools, such as CDF, box plots and histograms. This work presents only the 0 dB noise and 15 dB noise cases, which are shown for the first scenario in Figure 4, Figure 5, Figure 6 and Figure 7, but other graphs could also have been provided if desired. In the given figures, the 2s, 3s and 4s denote two sources, three sources and four sources, respectively. Figure 4, Figure 5, Figure 6 and Figure 7 show that the fitness value of two sources is less than the fitness value of three sources. In addition, the fitness value of three sources is less than the fitness value of four sources. It is because when the number of unknown values increases, the problem becomes more difficult; thus, the performance of the algorithm decreases slightly. 

For the second scenario, the same number of sources as the first scenario is taken into account, i.e., two sources, three sources and four sources. A different noise level is added to each of them in turn and in steps, i.e., first, two sources are considered and initially, the 0 dB noise is added to these two sources and the data are obtained for 100 runs of the proposed scheme FPA. Next, each set of data is obtained for 100 independent runs of the same proposed algorithm (FPA) for 5 dB, 10 dB, and 15 dB noise levels added to the same two sources, respectively. The same is carried out with the three sources and four sources cases. Table 3 shows the best estimated DOAs and their RMSE with different noise levels for two sources. Table 4 shows the estimated DOAs and their RMSE with different noise levels for three sources. Likewise, Table 5 shows the estimated DOAs and their RMSE with different noise levels for four sources. In each case, the desired DOAs considered are as follows:

$$\begin{array}{l} Desired\;DOAs\;for\;Two\;Sources=[-35\;\;\;\;35]\\ Desired\;DOAs\;for\;Three\;Sources=[-40\;\;\;\;40\;\;\;\;50]\\ Desired\;DOAs\;for\;Four\;Sources=[-55\;\;\;\;55\;\;\;\;65\;\;\;\;\;-65] \end{array}$$

Furthermore, the graphs of the best fitness values, CDF, box plots and histograms are shown in Figure 8, Figure 9, Figure 10 and Figure 11 for the second scenario. In Figure 10, the symbols 2sn0, 3sn0 and 4sno represent two sources, three sources and four sources with 0 dB noise, respectively. Likewise, 2sn5, 3sn5 and 4sn5 represent two sources, three sources and four sources with 5 dB noise, respectively, and so on. Figure 4a shows that the best fitness achieved for two sources with 0 dB noise is about 0.28 in 100 runs of the proposed algorithm. In the same graph, it is clear that the best fitness achieved by three sources is about 0.33 in 100 runs and likewise, the best fitness achieved by four sources in 100 runs is about 0.34. Figure 4b shows the graph for the 15 dB noise case that is added to the same two sources, three sources and four sources, respectively. It can be observed from this graph that as the noise is reduced from 0 dB to 15 dB, the fitness of all the sources (2s, 3s and 4s) is improved, i.e., the best fitness achieved in 100 runs by two sources is about 10^−2^. Likewise, the best fitness values achieved by three sources and four sources in the same 100 runs are less than 10^−2^. Figure 5 shows the CDF analysis for the same two noise levels 0 dB and 15 dB, which are added to the same two sources, three sources and four sources, respectively. Figure 5a shows that about 1% of runs of the proposed algorithm gives a fitness value of about 0.28 for two sources. Likewise, about 1% of runs give a fitness value of about 0.33 for three sources and about 1% of runs give a fitness value of 0.34 for four sources. Figure 5b shows the same sources with the 15 dB noise case. From this figure, it is clear that about 2% of runs give a fitness value of about 10^−2^ for two sources, but the same 2% of runs give a smaller fitness value from 10^−2^ for three sources and four sources, respectively. Figure 6 displays the box plot analysis of the same two cases, i.e., 0 dB and 15 dB noise, which are added to the same two sources (2s), three sources (3s) and four sources (4s), respectively. Figure 6a shows that the worst fitness achieved by two sources (2s) is about 0.42. Similarly, the worst fitness values achieved by three and four sources (3s and 4s) are about 0.47 and 0.48, respectively. Moreover, the best fitness values achieved by two sources (2s), three sources (3s) and four sources (4s) are about 0.36, 0.41 and 0.42, respectively. Fifty percent of the fitness values are less than 0.42 for two sources (2s), less than 0.44 for three sources (3s) and less than 0.45 for four sources (4s). Figure 6b shows that the worst fitness values achieved by two sources (2s), three sources (3s) and four sources (4s) are about 0.013, 0.020 and 0.030, respectively. Likewise, the best fitness values achieved by the same two sources, three sources and four sources are about 0.0122, 0.017 and 0.027, respectively. Fifty percent of the fitness values are less than 0.016 for two sources, less than 0.78 for three sources and less than 0.029 for four sources, respectively. As is clear from these figures, when the noise level decreases, the fitness values are also improved. One can verify very easily from these graphs that for 0 dB noise, the fitness value is higher than the case where the noise level is decreased to the 15 dB level. Likewise, Figure 8, Figure 9, Figure 10 and Figure 11 demonstrate that even for the lower values of SNR, the proposed algorithm performed well. In summary, all of these graphs show that the novel scheme FPA performs well in each scenario. 

**Part 2:** This part presents the comparison of our proposed FPA algorithm with the state-of-the art algorithms in the literature [18,19,20,21].

**Case 1:** In this case, the number of antennas in T_x_ and R_x_ is chosen to be N = 5 and M = 10, respectively. The inter-element distances between each two consecutive antennas in both T_x_ and R_x_ arrays are kept as the same, i.e., half the wavelength. Furthermore, 20 dB additive white Gaussian noise (AWGN) was added to the received signals for practicability purposes. Three targets are considered, of which the desired values of the DOA are considered to be −30, 30 and 50. The proposed scheme is run 100 times independently and a large amount of data is obtained. The performance of our proposed scheme FPA can be verified from Table 6, as it performed better compared to the other algorithms in terms of estimation accuracy and RMSE. The second-best result is achieved by [20].

In the same way, the proposed algorithm is further tested for considering a lower number of antennas in R_x_, i.e., M = 4. Although the results are slightly degraded, one can still verify from Table 7 that the proposed algorithm performed fairly well compared to the other algorithms.

**Figure 12:**
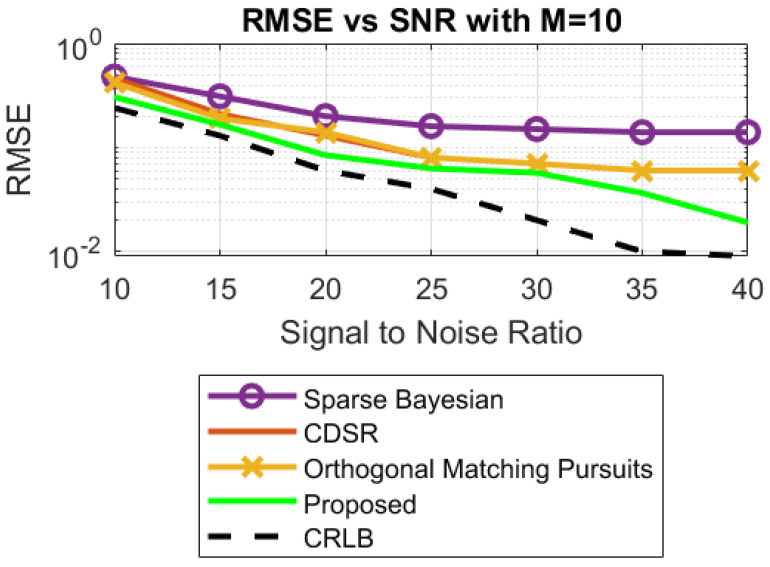
RMSE vs. SNR with M = 10.

**Figure 13:**
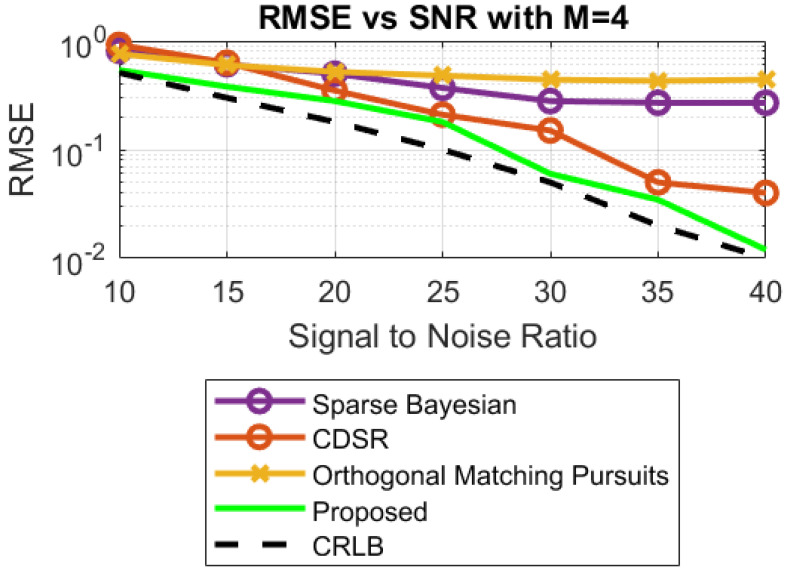
RMSE vs. SNR with M = 4.

**Case 2:** In this case, simulations are carried out to validate the robustness against the noise of the proposed algorithm FPA. Different values of SNRs are considered, which range from 10 dB to 40 dB. The RMSE of the proposed algorithm is minimal, as compared to its counterpart algorithms for R_x_ with M = 10 and M = 4, as shown in Figure 12 and Figure 13. Again, the second-best RMSE is produced by the algorithm discussed in [20]. 

The corresponding Cramer–Rao lower bound (CRLB) for the DOA estimation shown in Figure 12 and Figure 13 can be obtained from [21].

Case 3: In this case, the computational complexity of the proposed algorithm is compared with [18,19,20] for three targets. As provided in Table 8, the proposed algorithm requires 1.08 s to attain the desired outcomes, which is higher than the algorithms discussed in [18,19,20]. The best computational complexity is achieved by [18].

## 5. Conclusions and Future Work

In this work, the problem of DOA estimation for monostatic MIMO radars was considered. A novel and efficient approach based on flower pollination was designed and implemented. The proposed approach is inspired by the pollination process of flowering plants that has successfully solved different optimization problems. In the present work, the FPA was optimized by incorporating a new fitness function based on extended array manifold vectors. This fitness function works on the mean square error that defines an error between the estimated and the reference responses of the system. It required a single snapshot to attain the desired outcomes. The efficiency of the proposed algorithm was compared to the already existing methods in the literature and it has shown comparatively better results in terms of RMSE and estimation accuracy. However, the computational complexity of our proposed algorithm is slightly higher than the other algorithms. Different statistical tools such as CDF, box plots and histograms were used to verify the effectiveness of the proposed algorithm.

In the future, this approach can be used in the fields of adaptive beamforming, null steering, tracking multiple targets, etc. Future research into the suggested meta-heuristics computing approach may prove to be a good option for dependable, effective, and precise DOD/DOA estimation in various applications of utmost importance [33,34,35].

## Figures and Tables

**Table 1:** Parameter Settings of FPA.

S.No.	Name	Specification
1.	Population Size	10 members
2.	Probability Switch (PSW)	0.8
3.	Number of Iterations	2000
4.	Lower Bound	−90°
5.	Upper Bound	90°

**Table 2:** System Specifications.

S.No.	Name	Specification
1.	Operating System	Windows 10 Enterprise, 64 bit
2.	RAM	32 GB
3.	Processor	Intel(R) Core (TM) i7-7820HQ CPU @ 2.90 GHz 2.90 GHz
4.	Software	MATLAB R2022b

**Table 3:** DOA Estimation for Two Targets for N = 6 and M = 6 with Different Noises.

Noise	DOAs		RMSE
	$\theta_{1}$	$\theta_{2}$	
0 dB	−34.6106	35.3599	0.0318
5 dB	−35.2284	34.8185	0.0286
10 dB	−35.1221	35.0787	0.0136
15 dB	−35.1136	35.0413	0.0267

**Table 4:** DOA Estimation for Three Targets for N = 6 and M = 6 with Different Noises.

Noise	DOAs			RMSE
	$\theta_{1}$	$\theta_{2}$	$\theta_{3}$	
0 dB	−39.6713	41.1235	52.2910	0.1626
5 dB	−40.0724	40.8380	50.4058	0.0915
10 dB	−39.7974	40.1003	50.1806	0.0387
15 dB	−40.0801	39.9033	49.6680	0.0270

**Table 5:** DOA Estimation for Four Targets for N = 6 and M = 6 with Different Noises.

Noise	DOAs				RMSE
	$\theta_{1}$	$\theta_{2}$	$\theta_{3}$	$\theta_{4}$	
0 dB	−55.9432	54.0006	65.7209	−67.0888	0.1931
5 dB	−55.5708	55.3826	65.1077	−65.3172	0.1802
10 dB	−56.1675	54.1641	64.5014	−67.3498	0.1379
15 dB	−54.9222	54.6566	65.0776	−64.6327	0.0855

**Table 6:** DOA Estimation for Three Targets for N = 5 and M = 10 with SNR = 20 dB.

Scheme	$\theta_{1}$	$\theta_{2}$	$\theta_{3}$	RMSE
Desired DOA	−30	30	50	--
SBL Method [19]	−30.0260	29.8147	50.6640	0.1300
CDSR [20]	−30.0960	29.8947	50.1740	0.4082
OMP Method [18]	−30.0260	29.9447	50.7040	0.3985
Proposed Scheme	−30.0000	30.0000	50.0000	0.0901

**Table 7:** DOA Estimation for Three Targets for N = 5 and M = 4 with SNR = 20 dB.

Scheme	$\theta_{1}$	$\theta_{2}$	$\theta_{3}$	RMSE
Desired DOA	−30	30	50	-
SBL Method [19]	−31.0260	30.8147	50.1640	0.7009
CDSR [20]	−30.8060	30.8547	49.6940	1.0591
OMP Method [18]	−31.3860	31.0947	49.5040	0.7623
Proposed Scheme	−30.0366	30.0905	49.8765	0.0909

**Table 8:** Computational complexity.

Schemes	Computational Times (sec)
Sparse Bayesian [19]	1.0702
CDSR [20]	0.6037
OMP [18]	0.1051
Proposed	1.08

## Data Availability

Not applicable.
